# Prevalence of Systemic Arterial Hypertension in Quilombola Communities, State of Sergipe, Brazil

**DOI:** 10.5935/abc.20190143

**Published:** 2019-09

**Authors:** Deyse Mirelle Souza Santos, Beatriz Santana Prado, Cristiane Costa da Cunha Oliveira, Marcos Antonio Almeida-Santos

**Affiliations:** Universidade Tiradentes, Aracaju, SE - Brazil

**Keywords:** Cardiovascular Diseases, Hypertension, Prevalence, Public Health, Risk Group, African Continental Ancestry Group, Health of Specific Groups

## Abstract

**Background:**

The quilombolas are groups formed by black ancestry individuals, living in a context of social vulnerability due to low socioeconomic level, which influences health care and the development of chronic diseases.

**Objective:**

To assess the prevalence of systemic arterial hypertension and its association with cardiovascular risk factors in the quilombola population in the State of Sergipe, Brazil.

**Methods:**

Study design was cross sectional, involving the administration of a questionnaire to individuals aged ≥ 18 years, in 15 quilombola communities of the State of Sergipe, Brazil. A value of two-sided p < 0.05 was considered statistically significant.

**Results:**

sA total of 390 individuals were evaluated, 72.3% of whom were women, with a mean age of 44.7 years. The prevalence of hypertension was 26% (with a confidence interval of 95% [95% CI]: 22-30), with no significant sex-related differences. The age was associated with arterial hypertension (95% CI: 1.03-1.06), systolic (95% CI: 1.04-1.07) and diastolic (IC 95%: 1.01-1.04) arterial hypertension. The level of body mass index was associated with arterial hypertension (95% CI: 1.00-1.11) and diastolic arterial hypertension (95% CI: 1.03-1.17). Economic class was associated with diastolic arterial hypertension (95% CI: 1.22-5.03).

**Conclusion:**

The prevalence of arterial hypertension in the quilombola communities was high. Its association with cardiovascular risk factors indicates the need to improve access to healthcare services.

## Introduction

The quilombolas are groups formed by black ancestry individuals, due to their African origin, trafficked to Brazil between the XVI and XIX centuries. They were brought to work as slaves in the sugar plantations under precarious conditions. After the abolition of slavery, numerous quilombola communities arose in Brazil; nowadays there are 2,958 communities throughout the country, and 35 are located in the State of Sergipe. The States of Bahia, Maranhão, Pará, Minas Gerais and Pernambuco have a higher number of communities in their territories.^1^ The land demarcated as quilombola territories ensure the physical, social, economic and cultural reproduction of the remaining members of the Quilombo communities.^2^

The quilombola communities are inserted in a context of social vulnerability due to low socioeconomic level, which directly influences healthcare and the development of chronic diseases.^3^ Studies have shown that systemic arterial hypertension (SAH) is one of the most relevant diseases among the quilombola populations, and can be associated with genetic factors. However, Brazilian studies could not associate genetic polymorphism with increased blood pressure levels among the quilombolas, which may be associated with the intense Brazilian miscegenationa.^4,5^

The prevalence of SAH among quilombola communities has ranged from 38.4%^6^to 45.4%,^7^ which represents a higher percentage rate compared to the general Brazilian population.^8^ The risk factors for the development and grievance of arterial hypertension are diseases like dyslipidemia, abdominal obesity, glucose intolerance, diabetes mellitus (DM), in addition to modifiable factors, such as socioeconomic determinants and inadequate access to healthcare services.^9^ SAH can cause permanent damage to individuals through the onset of cardiovascular, cerebrovascular and kidney diseases.^10^

Thus, the aim of this study was to identity the prevalece of SAH and its association with cardiovascular risk factors in the quilombola population of the State of Sergipe, Brazil.

## Methods

### The study design and sample

This is a cross sectional study, carried out in quilombola communities in the State of Sergipe, Brazil, in the period between September 2016 and April 2017. The sample delineation was performed through random selection in the quilombola communities, using the existing proportion of the population in the communities. These data were provided by the National Institute of Colonization and Agrarian Reform (INCRA).^1^

A random sample of clusters was selected in two stage cluster sampling. There are 35 quilomobola communities registered in the State of Sergipe, distributed in eight territories, of which four were randomly selected. Out of these four territories, 15 communities were randomly selected from a total of 19. Between 15% and 20% of the adult population voluntarily participated. For each stage, once the territories and the quilombola communities were registered, the random sampling without replacement was performed using the Stata^®^ version 15.1 software.

The communities studied are far from the city headquarters, in areas of difficult acess. Certain communities (Resina and Pontal da Barra) surround the main river in the region and the sea, respectively. The other communities (Mocambo, Canta Galo, Pirangy, Terra Dura, Forte, Caraíbas, Bongue, Patioba, Ladeiras, Alagamar, Aningas and Quebra Chifre) are situated in large land properties. The quilombola community “Maloca” is the only one that is located in urban area among the other remaining communities in the state.^1^

The target population of the research, according to oficial registries,^1^ was estimated in 1,979 adult individuals, inhabitants of the 15 quilombola communities. Sample size calculations were done using the G*Power 3 software,^11^ respecting the following parameters: 80% power; two-sided alpha = 0.05; covariable distribution pattern; log-normal distribution; potential correlation between predictors, 0.80; expected prevalence of arterial hypertension in the general population (20.4%).^8^ According to these parameters, about 350 individuals would be necessary to detect an odds ratio ≥ 1.5 for differences between categorical predictors, in multiple regression logistic analysis. With the aim of preserving these characteristics in a potential situation of missing data, the sample size was increased to about 10%, totalling 390 individuals.

The inclusion criteria adopted for individual selection were: age ≥ 18 years; and being registered as quilombolas in the communities where they belong and in the INCRA. The exclusion criteria were: practice of physical exercise in the last 60 minutes; ingestion of alcoholic drinks, coffee or food; use of cigarette or consumption of other substances within the 30 minutes prior to blood pressure measurement; pregnancy; and amputated upper limbs.

### Clinical and Sociodemographic Data Collection

The data were collected using individual interview. The interviewers were trained for this procedure. The interview instrument used was a semi-estructured questionnaire adapted from the following studies: the Brazilian Ministry of Health’s Food Guide^12^ and the Evaluation of Physical Activity Program Effectivity in Brazil,^13^ both published by the Brazilian Ministry of Health; the National Household Sample Survey ;^14^ the criteria of economic classification of the ABEP (Brazilian Association of Market Research Firms), which divides society into economic classes A, B1, B2, C1, C2, D-E, considering household assets, education level and the public services available.^15^ The questions related with licit and illicit drugs were based on the Brazilian version of ASSIST (Alcohol, Smoking and Substance Involvement Screening Test)^16^ The previous history of diseases was based on the questions asked for admission to hospital due to primary care-sensitive conditions.^17^

Then, three blood pressure measurements were perfomed (with a 1-minute interval between each measurement). A Welch Allyn DuraShock™ DS44 (Welch Allyn, Curitiba, Brazil), internationally validated, Aneroid Sphygmomanometer, with nylon cuff and metal clasp, was used. The measurements were performed at the end of the interview.

During BP measurements, the individuals remained seated, with their legs uncrossed, feet flat on the floor, back supported by the back of a chair and relaxed. The individual’s left arm was positioned for measurement, followed by the right upper limb. The third measurement was performed on the limb that presented the highest value, always with the arm rested on a table, at heart level.

For analysis, the mean of the three measurements was calculated, which corresponded to the research criteria, being considered hypertensive those individuals who had systolic arterial pressure ≥ 140 mmHg and/or diastolic arterial pressure ≥ 90 mmHg.^9^ These more conservative measurements have been adopted because the three measurements of the blood pressure were performed in only one day. For this reason, the classification of the American Heart Association was not adopted.^18^

The Body mass (BMI) index [kg/m^2^] was estimated to evaluate the anthropometric measurements (weight and height). The BMI found was categorized according with the following measures: low weight, < 18.5 kg/m^2^; normal weight, 18.5 to 24.5 kg/m^2^; overweight, 25 to 29.9 kg/m^2^; level I obesity, 30 to 34.9 kg/m^2^; level II obesity, 35 to 39.9 kg/m^2^; and level III obesity, > 40 kg/m^2^.^19^

### Statistical analysis

Categorical variables were expressed as absolute numbers and percentage. The continuous variables were expressed as mean and standard deviation. To produce robust estimates independent from the distribution pattern of the variables, some tests were specifically adopted. sThe comparisons between continuous variables and two groups were performed using the unpaired student t-test with adjustment for heterogeneity of variance and degrees of freedom using the Satterthwaite method. Comparisons between continuous variables and more than three groups were estimated using the Kruskal-Wallis test. Several logistic regression models for AH were used, starting from the choice of predictors with p < 0.20 in unadjusted analyses. The model's potential increment was assessed after inclusion of squared terms and interaction of predictors. The comparison of the increased prevalence between the quilombola communities and the population in general was performed using the chi-squared adjustment test. To adjust the analysis for the differences between groups and the potential of heteroskedasticity in the quilombola communities, the Huber-White method was used to estimate clustering, robust standard errors, according with the 15 communities.

The estimate of the effect size was presented in odds ratio with 95% confidence interval. The Hosmer-Lemeshow test and C-statistics (area under the receiver operating characteristic curve, or ROC curve) were used to assess the potential calibration and discrimination of the model, respectively. A value of two-sided p < 0.05 was considered statistically significant and the Stata^®^ version 15.1 software (Stata Corp, College Station, TX, EUA), was used for data analysis.

## Results

A total of 408 volunteers participated in the research; out of these, 18 were excluded: four of them who reported being pregnant, and 14 because they had consumed alcohol. A total of 390 individuals were deemed eligible, 72.3% women and 27.7% men. There were no missing data. The age ranged from 18 to 101 years, with a mean equal to 44.7 ± 19 years. The skin color was self-reported, according to the criteria of the Brazilian Institute of Geography and Statistics (IBGE), which indicated that 50% of the individuals were brown-skinned. The most prevalent level of education was illiterate/incomplete primary education I (58%). In the economic field, classes D and E obtained greater representation (76.41%). Table 1 presents the frequency of the main sociodemographic characteristics of the quilombola communities studied.

**Table 1:** Distribution of the demographic and socioeconomic variables in quilombola comunities in the State of Sergipe, Brazil, 2016-2017

Variables	N	%
**Age**		
18 to 49	245	63
50 to 79	133	34
> 80	12	3
**Sex**		
Female	282	72.31
Male	108	27.69
**Skin Color/Race**		
Black	150	38.46
Brown	209	53.59
White	31	7.95
**Level of Education**		
Illiterate/Incomplete Primary Education I	226	58
Complete Primary Education I/Incomplete Primary Education II	64	16.43
Complete Primary Education II/Incomplete High School	50	12.83
Complete High School/Incomplete Higher Education	45	11.54
Complete Higher Education	5	1.20
**Economic classification**		
B2	5	1.28
C1	18	4.62
C2	69	17.69
D-E	289	76.41

A prevalence of 26% (95% CI: 22-30) was observed for SAH; systolic arterial hypertension in 22% (95% CI: 18-26) and diastolic arterial hypertension in 16% (95% CI: 12-20) of the cases. A chi-square test was performed to compare the prevalence of SAH in the quilombola communities and in the general population of Sergipe (20.4%),^8^ and the quilombola communities had a significantly higher prevalence (p = 0.0071).

The mean number of years with a previous SAH diagnosis was 9.59 (standard deviation = 8.66). The diagnosis of the disease had been made at a minimum age of 18 years and at a maximum age of 55 years.

There was no significant sex-related differences between the subclassifications of blood pressure. In women the average value of systolic pressure was 125.35 mmHg (95% CI: 122.7-127.9), whereas in men the average value was equal to 129.53 mmHg (95% CI: 125.3-133.7); *p* = 0.09. The average diastolic pressure value estimated for women was 78.88 mmHg (95% CI: 77.1-80.6); as for men, the average value was 78.57 mmHg (95% CI: 76.3-80.7); p = 0.83.

Among the behavioral variables reported by the participants, the following percentages were obtained: smoking, 37.18%; having alcohol drinking habits, 60.77%; and being physically inactive, 44.10%.

The participants responded that they consumed high quantities of sodium chloride (salt) everyday (17.69%). In relation to the anthropometric parameters, about 60.01% of the population presented with overweight or classes I, II and III obesity, with a smaller number of normal weight individuals (37.17%) (Table 2).

**Table 2:** Distribution of behavioral variables, lifestyle, anthropometric profile and risk fators in quilombola communities in the State of Sergipe, Brazil, 2016-2017

Variables	N	%
**Smoking**		
Yes	145	37.18
No	245	62.82
**Alcohol consumption**		
Yes	237	60.77
No	153	39.23
**Dyslipidemia**		
Yes	71	18
No	318	82
**Diabetes Mellitus**		
Yes	36	9.23
No	354	90.77
**Physical activity**		
Light	172	44.10
Moderate	77	19.74
Vigorous	141	36.16
**Fatty food consumption**		
< 1 time/week	130	33.33
1 or 2 times/week	113	28.98
3 or 4 times/week	147	37.69
**Candy consumption**		
< 1 time/week	185	47.44
1 or 2 times/week	108	27.69
3 or 4 times/week	97	24.87
**Daily intake of high-sodium foods**		
Yes	69	17.69
No	321	82.31
**Adds salt to served food**		
Yes	49	12.56
No	341	87.44
**Body mass index categories**		
Underweight	11	2.82
Normal weight	145	37.17
Overweight	139	35.64
Classes I, II and III obesity	95	24.37

In the univariate logistic regression analysis, the risk factors associated with arterial hypertension were: smoking (p = 0.02) and BMI (p = 0.04). In the multivariate analysis, the the odds ratio with statistical significance for arterial hypertension were found for the predictors age and BMI. For systolic arterial hypertension alone, the only significant statistic preditor was age; as for diastolic arterial hypertension, the predictors were age, BMI and, primarily, economic class (Table 3).

**Table 3:** Predictors of systemic arterial hypertension in quilombola communities in the State of Sergipe, Brazil, 2016-2017

Variables	AH			SAH			DAH		
	OR	95% CI	p	OR	95% CI	p	OR	95% CI	p
Age	1.05	1.03-1.06	< 0.001	1.06	1.04-1.07	< 0.001	1.02	1.01-1.04	< 0.001
**Sex**									
Female (ref.									
Male	0.71	0.38-1.33	0.29	0.67	0.36-1.24	0.24	0.98	0.48 -2.01	0.97
**ABEP**									
B2-C2 (ref.)									
D-E	1.75	0.93-3.28	0.07	1.38	0.74-2.56	0.29	2.47	1.22-5.03	0.01
BMI	1.05	1-1.11	0.04	1	0.95-1.05	0.84	1.10	1.03 -1.17	0.02

AH: arterial hypertension (systolic, diastolic or both); SAH: systolic arterial hypertension (alone); DAH: diastolic arterial hypertension (alone); OR: odds ratio; CI: confidence interval; ABEP: Brazilian Association of Market Research Firms; BMI: body mass index.

**Figure 1:**
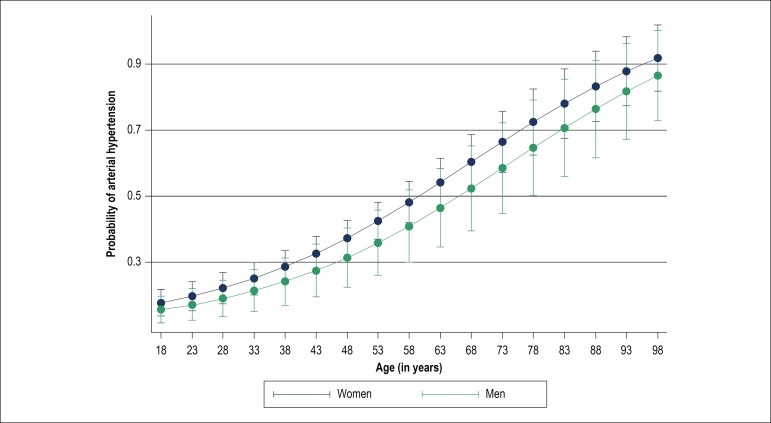
Probability of arterial hypertension in Quilombola communities according to age and sex.

The logistic regression model enabled us to identify the probability of developing arterial hypertension through increased BMI. Among the sexes, the number of women was higher. According with the age and sex, it was noticeable that, as the quilombola population grows older, the number of hypertensives tends to increase, especially among women (Figure 1).

The Hosmer-Lemeshow test showed good adjustment/calibration of the final model (p = 0.14). To assess the discrimination capacity of the model, the C-statistics was performed through calculation of the area under the ROC curve, presenting a value equal to 0.77, which was considered a satisfactory value.

## Discussion

The prevalence of SAH in the quilombola communities in the State of Sergipe (26%) was high, when compared with the estimates of the population in general (20.4%) in the same State,^20^ in similar age ranges.

In accordance with other studies in the general population developed in Brazil^21^ and in other multiracial countries,^22,23^ the prevalence of SAH was associated with increased age. The black ethnicity showed a higher predisposition to arterial stiffness than the other ethnicities.^24,25^

aAlthough the prevalence of arterial hypertension was higher when compared to the general population, our results found a lower prevalence than other studies.^6,7^ This difference may be due to methodological issues (such as the number of measurements and the conditions under which they were performed), regional variations (for example, alcohol consumption and sodium ingestion) or even ethnic issues which remain unclear, beyond the scope of this study.

In this study, no significant sex-related differences were observed in the occurrence of SAH or its subclassifications (systolic and diastolic) among the quilombolas. This data stands in contrast to what is found in the literature in the context of the general population^26^ and the quilombola population.^27^

Concerning the modifiable variables, increased BMI was one of the major predictors associated with arterial hypertension. Cross sectional studies have shown such association and the damage to the health of the quilombola population,^3^ whose inadequate lifestyle choices may be a result of low income and education.^28^

The prevalence of physical inactivity is this study was high. Probably, the idleness in rural areas promotes physical inactivity for most part of the months, when it is not harvest or planting time. This data corroborates with researches developed in rural^29^ and quilombola^30^ populations. This fact may have contributed for obesity and physical inactivity to foster the onset of arterial hypertension in the quilombola communities studied here.

It should be stressed that, when salt consumption was measured, low salt intake in this population may not have been accurately asessed, since sodium intake through processed or ultra-processed foods consumed everyday was not taken into consideration.^31^

The observed association between smoking and hypertension was significant in this study, which corroborates the results of other population-based studies.^27,32^ Another important data was alcohol consumption, which showed a high prevalence. However, this factor was not associated with arterial hypertension, corroborating the results of other studies developed in the quilombola communities.^21,30^

Among the limitations of this research, we can mention the fact that the participants were volunteers, that is, the communities were randomly selected and the sample size was determined in advance, but the the enrolment was voluntary. In addition, part of the male population was not accessible, because they were working in the fields or fishing when the visits took place. The presence of diabetes and dyslipidemia has not been investigated, since glucose and lipid measurements, respectively, were not performed and the mere response of the individuals enrolled was avoided, because it could lead to biased information.

Future research should adequately assess these risk factors among the quilombolas to obtain better comprehension, since, as far as we know, this is the first study to approach this issue in the quilombola communities of the State of Sergipe.

## Conclusion

The prevalence of arterial hypertension among the quilombolas was higher than in the general population. Age and increased BMI were the major predictors. This finding sugests the need for greater health care for the quilombolas, and serves as a baseline for the Brazilian government’s development of health strategies in line with the the needs of ethnoracial communities.
